# IFNγ Is a Key Link between Obesity and Th1-Mediated AutoImmune Diseases

**DOI:** 10.3390/ijms22010208

**Published:** 2020-12-28

**Authors:** Heekyong R. Bae, Myung-Sook Choi, Suntae Kim, Howard A. Young, M. Eric Gershwin, Seon-Min Jeon, Eun-Young Kwon

**Affiliations:** 1 Omixplus, LLC., Gaithersburg, MD 20885, USA; bae@omixplus.com (H.R.B.); skim@omixplus.com (S.K.); 2 Laboratory of Cancer Immunometabolism, Cancer and Inflammation Program, Center for Cancer Research, National Cancer Institute-Frederick, Frederick, MD 21701, USA; younghow@mail.nih.gov; 3 Department of Food Science and Nutrition, Center for Food and Nutritional Genomics Research, Kyungpook National University, Daegu 41566, Korea; mschoi@knu.ac.kr; 4 Division of Rheumatology, Allergy and Clinical Immunology, University of California at Davis, Davis, CA 95616, USA; megershwin@ucdavis.edu; 5 R&D Center, APtechnologies Corp., Gyeonggi-do, Hwaseong-si 18469, Korea

**Keywords:** IFNγ, obesity, autoimmune diseases, Th1, luteolin, high-fat diet, RNA-seq, microarray, bioinformatics, animal study

## Abstract

Obesity, a characteristic of metabolic syndrome, is also associated with chronic inflammation and the development of autoimmune diseases. However, the relationship between obesity and autoimmune diseases remains to be investigated in depth. Here, we compared hepatic gene expression profiles among high-fat diet (HFD) mice using the primary biliary cholangitis (PBC) mouse model based on the chronic expression of interferon gamma (IFNγ) (ARE-Del^-/-^ mice). The top differentially expressed genes affected by upstream transcriptional regulators IFNγ, LPS, and TNFα displayed an overlap in HFD and ARE-Del^-/-^ mice, indicating that obesity-induced liver inflammation may be dependent on signaling via IFNγ. The top pathways altered in HFD mice were mostly involved in the innate immune responses, which overlapped with ARE-Del^-/-^ mice. In contrast, T cell-mediated signaling pathways were exclusively altered in ARE-Del^-/-^ mice. We further evaluated the therapeutic effect of luteolin, known as anti-inflammatory flavonoid, in HFD and ARE-Del^-/-^ mice. Luteolin strongly suppressed the MHC I and II antigen presentation pathways, which were highly activated in both HFD and ARE-Del^-/-^ mice. Conversely, luteolin increased metabolic processes of fatty acid oxidation and oxidative phosphorylation in the liver, which were suppressed in ARE-Del^-/-^ mice. Luteolin also strongly induced PPAR signaling, which was downregulated in HFD and ARE-Del^-/-^ mice. Using human GWAS data, we characterized the genetic interaction between significant obesity-related genes and IFNγ signaling and demonstrated that IFNγ is crucial for obesity-mediated inflammatory responses. Collectively, this study improves our mechanistic understanding of the relationship between obesity and autoimmune diseases. Furthermore, it provides new methodological insights into how immune network-based analyses effectively integrate RNA-seq and microarray data.

## 1. Introduction

Interferon gamma (IFNγ), the only characterized Type II interferon, is an inflammation modulator regulating both pro- and anti-inflammatory responses. IFNγ is predominantly produced by natural killer (NK) cells and natural killer T cells during the innate immune response and by CD4+ T helper (Th1) cells and CD8+ cytotoxic T cells in the humoral immune response. Furthermore, emerging evidence shows that other cell types, including macrophages, dendritic cells, and neutrophils, also produce IFNγ in a context-dependent manner. IFNγ expression is critical for the initial host innate immune response and is also essential to mount an adaptive immune response. Several reports have shown that serum IFNγ is increased in patients with autoimmune diseases such as systemic lupus erythematosus (SLE) and rheumatoid arthritis (RA) [1]. Corresponding to this data, *IFNG* polymorphisms are associated with susceptibility to these diseases in patient cohorts [2,3]. Aberrant IFNγ expression was observed in other autoimmune disease murine models, including experimental autoimmune encephalomyelitis and collagen-induced arthritis, in both protective and detrimental roles [4].

Primary biliary cholangitis (PBC) is an autoimmune disease of the liver, predominantly affecting middle-aged women but having a worse prognosis in men, which eventually leads to liver failure [5]. PBC is characterized by progressive destruction of the bile ducts in the liver, caused by autoreactive T cell-mediated self-attack on the biliary epithelial cells. Incidence of PBC is gradually increasing worldwide, but there is as of yet no cure, leaving liver transplantation as the sole treatment option in later stages. Previously, we reported that a mouse model (ARE-Del^-/-^), characterized by chronic expression of IFNγ, has strong serological, and pathological phenotypes of PBC [6]. These include portal inflammation, bile duct damage, positive anti-mitochondrial antibodies (AMAs, >95% positive in patients with PBC), and increased serum bile acid with disease progression. This mouse model was noted to be a powerful new research tool, as it strongly reflected clinical features of female-prevalent autoimmune diseases [7,8]. ARE-Del^-/-^ mice with chronic low expression of IFNγ are generated by genetic deletion of the AU-rich element (ARE) in the 3’-UTR region of *IFNG*. Given that PBC is thought to be a Th1-mediated autoimmune disease, our findings support a primary role for IFNγ in the development of PBC.

Over the last few decades, obesity has become a major research interest due to its dramatic increase worldwide and the clinical and economic burden of obesity-related diseases. Obesity-related inflammatory responses are associated with adipokines secreted by adipose tissue, which function as hormones and cytokines to modulate inflammatory responses and metabolic processes [9]. Recent emerging views indicated a potential relationship between obesity and major autoimmune diseases such as SLE, RA, multiple sclerosis, and type 1 diabetes [10,11,12,13]. However, the underlying mechanisms that connect these conditions remain to be elucidated. Leptin is an adipokine, primarily secreted by adipocytes, and is considered a key regulator of inflammatory responses and metabolic processes [14]. Here we characterize the central pathogenic role of IFNγ in HFD-induced inflammatory responses. Our data provide new insight into the mechanism of the interaction between IFNγ and leptin signaling, which furthers our understanding of the relationship between obesity and the development of Th1-mediated autoimmune diseases.

## 2. Results

### 2.1. Comparison of Top Regulators and Pathways between High-Fat Diet (HFD) and ARE-Del^-/-^ mice

**Figure 1:**
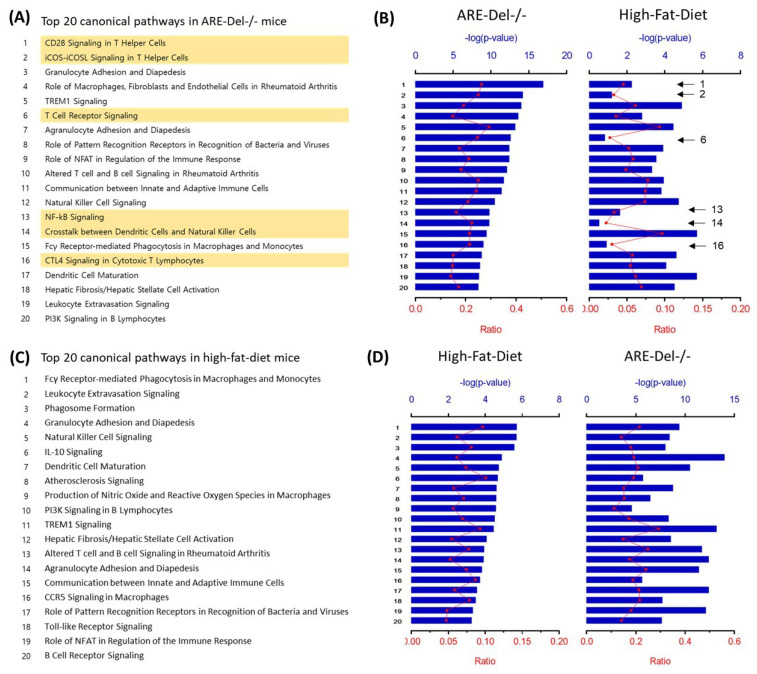
The comparison of altered pathways between high-fat-diet and ARE-Del^-/-^ mice. (**A**) The top 20 altered pathways in ARE-Del^-/-^ mice. Yellow marks represent pathways specifically increased in ARE-Del^-/-^ mice compared tohigh-fat-diet mice; (**B**) Overlapping pathways of high-fat-diet mice were aligned with those in (A); (**C**) The top 20 altered pathways in high-fat-diet mice; (**D**) Overlapping pathways of ARE-Del^-/-^ mice were aligned with those in (**C**). Ratio (bottom y-axis, red line) refers to the number of genes from the dataset divided by the total number of genes that make up that pathway from within the ingenuity pathway analysis (IPA) knowledge base. The -log of the *p*-value (top y-axis, bar) was calculated by Fisher’s exact test.

We compared the top upstream transcriptional regulators in both HFD and ARE-Del^-/-^ mice. The top upstream regulators in HFD mice were determined to be IFNγ (*p* = 9.19 × 10^−24^), TLR4 (*p* = 5.43 × 10^−23^), and TNF (*p* = 1.04 × 10^−21^) (Table 1). These transcriptional regulators overlapped with those in ARE-Del^-/-^ mice, as previously reported [6]. These data indicate that IFNγ signaling plays a critical role in HFD-induced inflammatory responses. We further characterized top pathways altered in HFD mice, and compared these pathways in ARE-Del^-/-^ mice (Figure 1). The top 20 altered pathways in ARE-Del^-/-^ mice are listed in Figure 1A. The top differential pathways in HFD mice were aligned and compared with those in ARE-Del^-/-^ mice (Figure 1B). It was notable that T cell-mediated responses were mostly detected in ARE-Del^-/-^ mice, but not in HFD mice. In the same way, we aligned the altered pathways in ARE-Del^-/-^ mice according to the top 20 canonical pathways in HFD mice (Figure 1C,D). These data show that innate immune responses were among the top pathways altered in HFD mice (Figure 1C), which were also strongly activated in ARE-Del^-/-^ mice (Figure 1D). Overall, there were robust similarities among the top upstream regulators and pathways between HFD and ARE-Del^-/-^ mice, suggesting that a HFD can aggravate the pathological development of PBC. Importantly, HFD mice display IFNγ-induced early innate inflammatory responses, whereas ARE-Del^-/-^ mice display more advanced adaptive immune responses.

### 2.2. The Therapeutic Effect of Luteolin on the Early Development of PBC

In a previous study, luteolin improved insulin resistance and hepatic steatosis by suppressing hepatic lipogenesis and lipid absorption in a HFD mouse model [15]. Given that IFNγ-mediated immune responses play a primary role in HFD-induced inflammatory responses, we further characterized the immune responses in HFD mice by treating these mice with luteolin. Luteolin is a natural flavonoid present in various fruits and vegetables, and many studies have shown that it possesses anti-oxidant, anti-cancer, and anti-inflammatory effects [16]. However, it is not yet understood how luteolin modulates immune responses in terms of these therapeutic effects. In Table 2, we compared the top 20 altered pathways in HFD mice with those altered after luteolin treatment and ARE-Del^-/-^ mice. Although similar pathways were activated in HFD and ARE-Del^-/-^ mice, luteolin downregulated these pathways in HFD mice, implying that it may also suppress early inflammatory responses in ARE-Del^-/-^ mice.

### 2.3. Luteolin Modulates Antigen Presentation and Energy Metabolic Pathways

**Figure 2:**
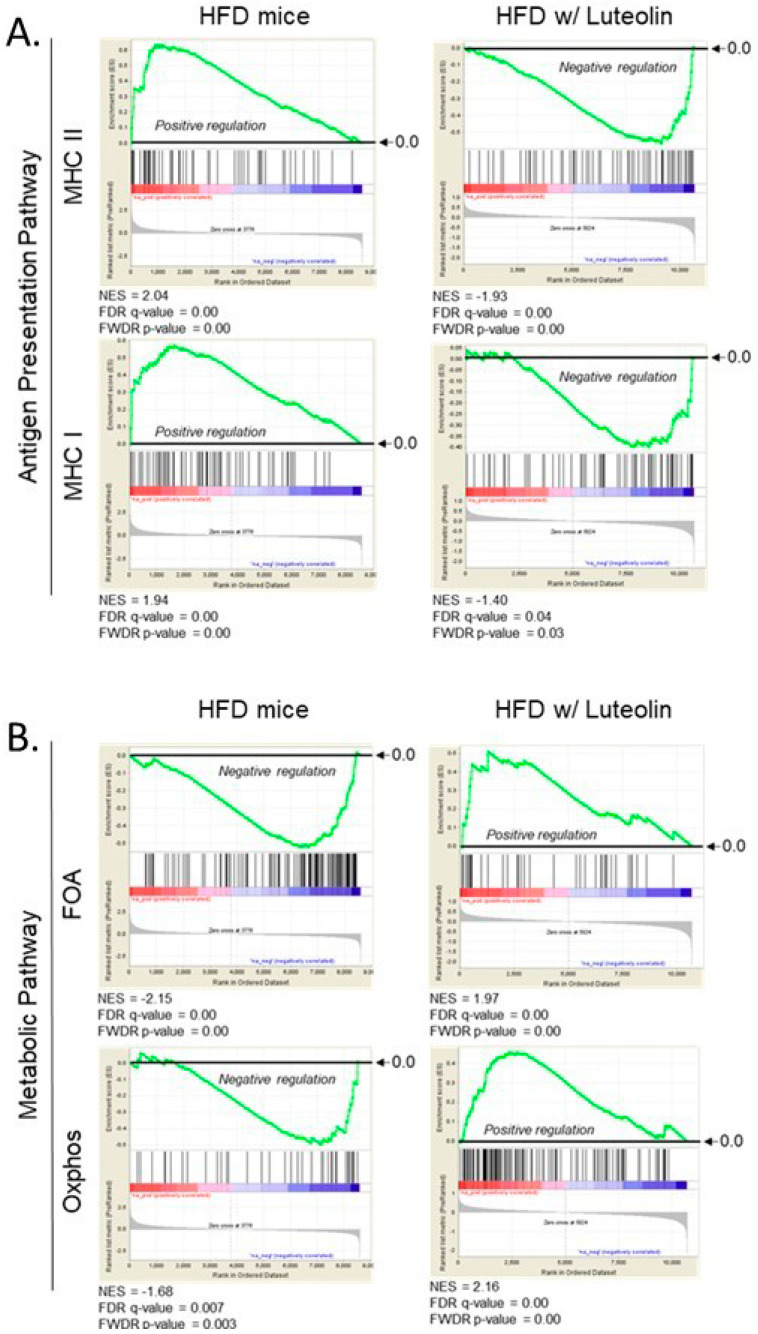
Gene Set Enrichment Analysis (GSEA) of antigen presentation pathways (**A**) and metabolic pathways (**B**) in high-fat-diet mice with/without luteolin treatment. (**A**) Expression of MHC-I and MHC-II gene sets in high-fat-diet mice with and without luteolin treatment; (**B**) Expression of FAO and Oxphos gene sets in high-fat-diet mice with and without luteolin treatment. FDR, false discovery rate; FWER, family wise error rate; NES, normalized enrichment scores.

We previously reported that chronic expression of IFNγ activated the antigen presentation pathway in the liver [6]. This indicates that early inflammatory response in ARE-Del^-/-^ mice also had a strong induction of antigen presentation pathways, including increased expression of MHC class I and II molecules. Consistent with this, gene set enrichment analysis (GSEA) profiles showed that HFD mice displayed positive regulation of MHC I and II expression, while these expressions were suppressed upon luteolin treatment (Figure 2A). Given that IFNγ is critical for antigen presentation in innate immune responses, and IFNγ is also important for primary inflammatory signaling in HFD mice, this result suggests that luteolin ameliorates inflammatory responses in the innate immune system, possibly via suppression of IFNγ signaling pathway.

We also analyzed changes in metabolic pathways, such as fatty acid oxidation (FAO) and oxidative phosphorylation (Oxphos). Emerging evidence suggests that immune cells such as macrophages and dendritic cells switch metabolic pathways to modulate their pro- as well as anti-inflammatory responses [17,18]. HFD mice displayed negative regulation of the FAO and Oxphos pathways as determined by hepatic gene expression (Figure 2B). At the same time, FAO and Oxphos pathways were strongly induced in HFD mice upon luteolin treatment. Even though these data represented changes in global hepatic gene expression in HFD mice, important information can still be gleaned about the potential effects of luteolin on the metabolic switch in immune cells.

### 2.4. HFD Dysregulates Macrophage Function, Possibly via the IFNγ Signaling Pathway

**Figure 3:**
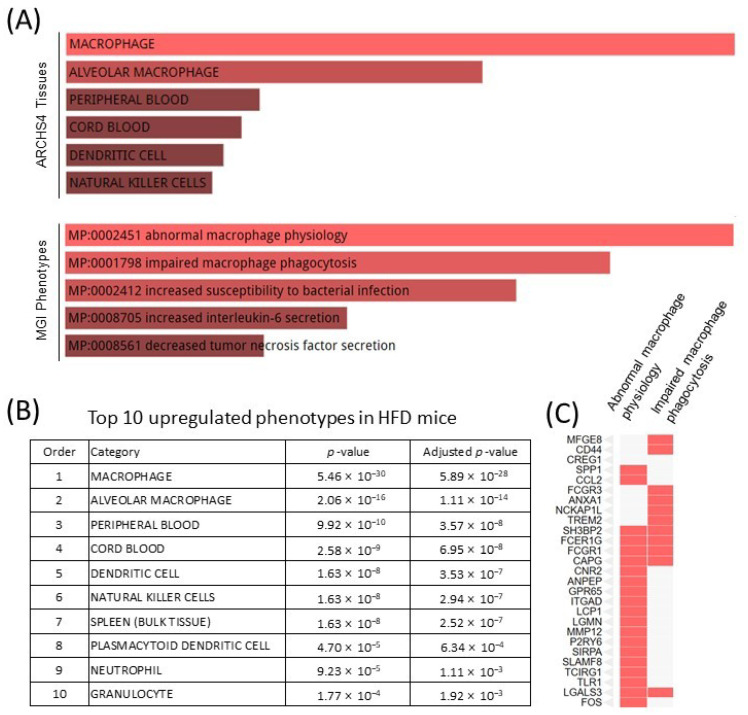
Pathway analysis of upregulated genes in the liver of HFD mice. (**A**) Abnormal macrophage function predicted by pathway analysis based on ARCH4 and MGI phenotypes databases; (**B**) The top 10 upregulated phenotypes according to the ARCH4 database; (**C**) A cluster graph of gene expression profiles of abnormal macrophage physiology and impaired macrophage phagocytosis in the MGI phenotypes database.

There were 269 upregulated genes in the liver of HFD mice, of which macrophages are one of the top cell phenotypes, according to the ARCHS4 database and Mouse Genome Informatics (MGI) database resources, and as characterized by Enrichr pathway analysis (Figure 3A). Other characterized cell phenotypes included innate immune cells, such as dendritic cells, plasmacytoid dendritic cells, and neutrophils (Figure 3B). These data support the previous conclusion that HFD-induced inflammation is involved in the innate immune response, particularly via macrophage immunomodulation. Notably, the HFD increased dysregulated macrophage function, including abnormal macrophage physiology and impaired macrophage phagocytosis according to the MGI database resources (Figure 3C). This is consistent with our previous report that IFNγ altered macrophage function and impaired autophagy [19], which can lead to impaired macrophage phagocytosis. These data reveal that the IFNγ signaling pathway in HFD mice plays a critical role in macrophage function.

### 2.5. Luteolin Activated Nuclear Receptor (NR) Signaling Pathways, Including LXR, RXR, and PPAR

**Figure 4:**
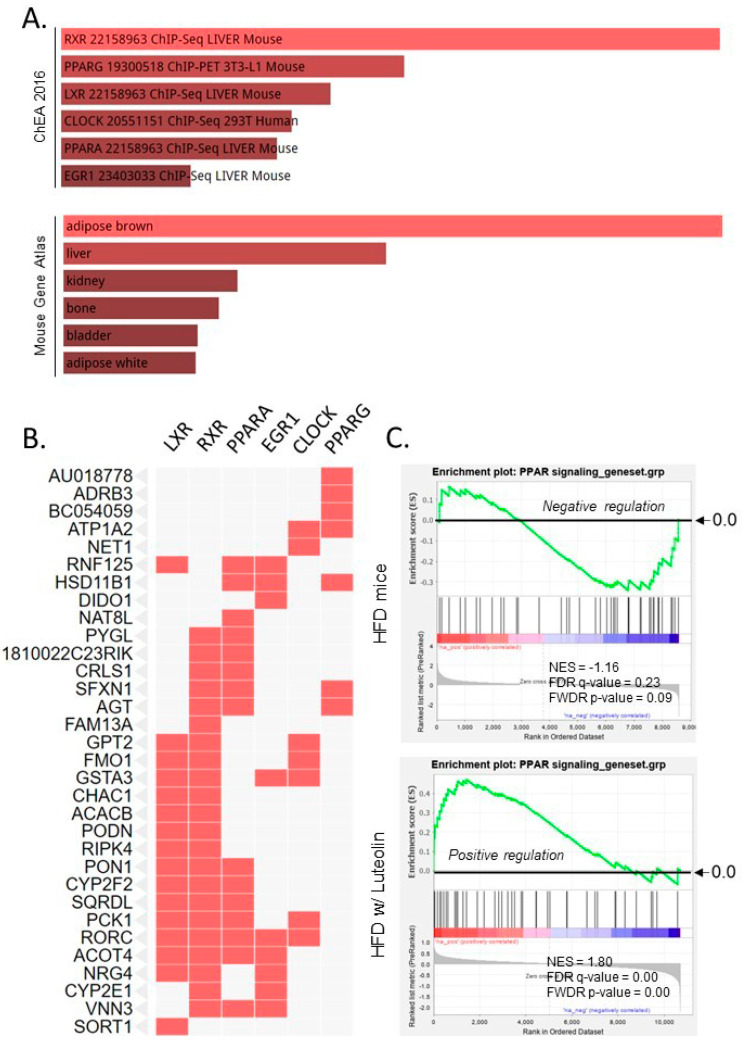
Pathway analysis of downregulated genes in the liver of HFD mice. (**A**) Downregulated NR signaling pathways and genes associated brown adipose tissue based on ChEA and Mouse Gene Atlas database; (**B**) A cluster graph of gene expression profiles of downregulated NR signaling according to ChEA database; (**C**) GSEA profiles of PPAR signaling in HFD with and without luteolin treatment.

Given that NR signaling, including the LXR, RXR, and PPAR signaling pathways, were suppressed in ARE-Del^-/-^ mice [19], we further analyzed 64 downregulated genes in the liver of HFD mice. The top pathways associated with these significantly downregulated genes were defined according to the Chromatin Immunoprecipitation (ChIP) Enrichment Analysis (ChEA; 2016) and Mouse Gene Atlas databases (Figure 4A). Interestingly, our data indicated that brown adipocyte gene expression was significantly downregulated (*p* = 1.9 × 10^−8^) in the liver of HFD mice, correlating with downregulated nuclear RXR, PPARγ, LXR, CLOCK, PPARα, and EGR1 signaling. The related genes in these NR signaling pathways are presented in a cluster graph in Figure 4B. Since luteolin is known to be a partial antagonist of PPARγ [20], we further compared PPAR signaling in HFD mice to HFD mice treated with luteolin using GSEA Pre-ranked analysis (Figure 4C). The GSEA profiles showed that the expression pattern of PPAR signaling was downregulated in the liver of HFD mice. In contrast, luteolin treatment significantly upregulated genes related to the PPAR signaling pathway in the liver of HFD mice, supporting that luteolin may be an agonist of both PPARα and PPARγ.

### 2.6. Interaction between IFNγ and Obesity-Related Genes

**Figure 5:**
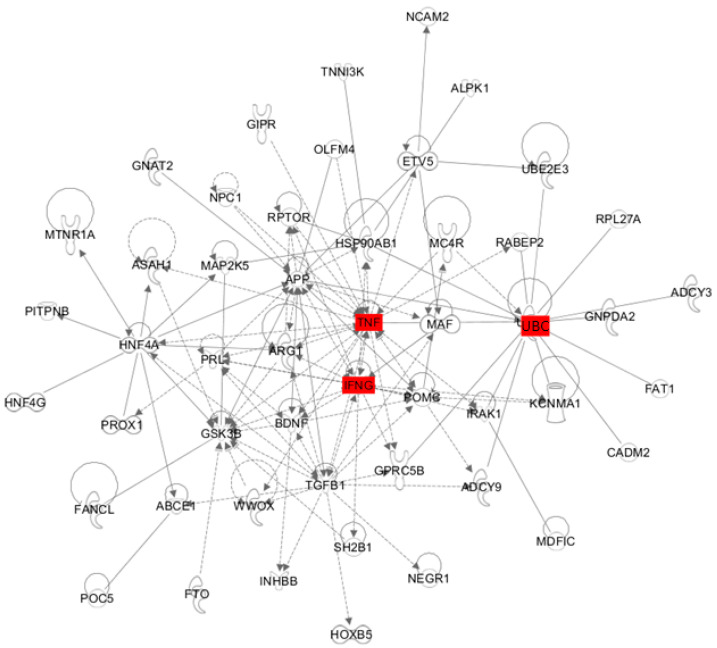
Interconnected gene networks between IFNγ and the most significant obesity-related variants. The most significant 55 variants were selected by the National Human Genome Research Institute GWAS catalog (*p* ≤ 1 × 10^−5^). *TNFA* and *UBC* were identified by explorative analysis in IPA. Solid and dashed lines indicate direct and indirect interactions, respectively.

To characterize the genetic interaction between IFNγ and obesity-related genes, we identified the 53 most significant variants (*p* ≤ 1 × 10^−5^), selected by the National Human Genome Research Institute GWAS catalog. Among these genes, 47 have known direct or indirect interactions with IFNγ (Figure 5). Using the pathway explorative analysis tool of ingenuity pathway analysis (IPA) software, we identified two genes, *TNFA* and *UBC* (Ubiquitin C), that maximize the connection between obesity-related genes and IFNγ. As shown in Table 1, TNFα signaling was one of top upstream transcriptional regulators in the liver of HFD mice, implying that IFNγ- and TNF-mediated inflammatory responses are major events in obesity-associated chronic inflammation. Although the functional role of UBC has not been well characterized because deletion of this gene results in embryonic lethality due to its requirement for fetal liver development [21], the genetic deletion of *UBB*, another gene encoding mammalian ubiquitin (Ub) protein, induced adult-onset obesity in mice [22]. This data yields new clues as to the potential role of UBC in obesity and obesity-associated inflammatory responses.

## 3. Discussion

Excessive lipid accumulation in obesity has been linked to the release of detrimental cytokines and chemokines that result in metabolic dysregulation. In obese humans with a body mass index over 30, pro- and anti-inflammatory cytokines are upregulated, including IL-5, IL-10, IL-12, IL-13, IFNγ, and TNFα [23]. Although obesity is known to have a component of low-grade, systemic inflammation, the relationship between obesity-associated inflammation and autoimmune diseases remains to be elucidated. The mouse model of autoimmune diseases used here displays chronic expression of IFNγ via the genetic modification of the AU-rich element in the 3′-UTR of *IFNG* mRNA [24]. Notably, the top upregulated transcriptional regulators in the liver of both HFD and ARE-Del^-/-^ mice displayed an overlap, suggesting that IFNγ may be a primary cytokine in obesity-associated inflammatory responses. These data also imply that the chronic expression of IFNγ in obesity aggravates Th1-mediated autoimmune diseases. In this analysis, we compared male HFD mice to female ARE-Del^-/-^ mice. As reported previously, ARE-Del^-/-^ mice have female-biased pathological and serological phenotypes of PBC, and the prevalence in females is a hallmark of major autoimmune diseases [25]. Considering that female mice have a strong sensitivity to Th1-mediated inflammatory responses, we hypothesized that female HFD mice would have more interesting pathological phenotypes compared to male HFD mice. Collectively, our data support the primary role for IFNγ in linking obesity to the development of autoimmune diseases.

There are remarkable differences in the upregulated inflammatory responses between HFD and ARE-Del^-/-^ mice, even though IFNγ is a common primary regulator in these conditions. HFD mice display early IFNγ-mediated innate inflammatory responses, whereas ARE-Del^-/-^ mice have greater adaptive immune responses, such as activation of T helper cells and associated signaling pathways. In ARE-Del^-/-^ mice, the low but chronic expression of IFNγ induced distinct pathological phenotypes similar to PBC [6], and early IFNγ-mediated inflammation was involved in the activation of the innate immune system, triggering antigen presentation signaling pathways [6]. In HFD mice, the low but chronic expression of IFNγ may stimulate early innate immune responses similar to those in ARE-Del^-/-^ mice, depending on the local and systemic levels of IFNγ. As previously reported, beyond a certain threshold, IFNγ breaks the immune tolerance which induces autoreactive B and T cells to attack self-epitopes in specific cells and organs, leading to autoimmune diseases [19]. Therefore, obesity-induced IFNγ signaling can be aggravated by infectious, genetic and environmental factors to break this immune tolerance barrier in the host immune system.

In adipocytes, IFNγ inhibits PPARγ-mediated gene expression by not only suppressing protein synthesis, but also increasing protein degradation of PPARγ [26]. The polyubiquitination of PPARγ is regulated by IFNγ pathways dependent on serine phosphorylation of PPARγ. Therefore, IFNγ regulates PPARγ expression, while PPARγ also directly regulates IFNγ expression. The PPARγ agonist thiazolidinedione rosiglitazone (Ro) suppresses expression of IFNγ target genes in macrophages [27]. Conversely, the PPARγ antagonist GW9662 increases lymphocyte IFNγ expression, and PPARγ ligands modulate expression at the IFNγ promotor, indicating that PPARγ negatively regulates the IFNγ promoter [28]. Collectively, these data suggest that PPARγ and IFNγ reciprocally suppress their activities, which may be a central regulatory node to modulate lipid metabolic pathways and Th1-mediated inflammatory responses.

Obesity increases the infiltration of macrophage in adipose tissue, and induces secretion of several inflammatory mediators [29]. Increased expression of IFNγ and TNFα are classified as markers of pro-inflammatory (M1) rather than anti-inflammatory (M2) polarization in macrophages [30]. Given that IFNγ and TNFα were determined to be top transcriptional regulators in HFD mice, it is likely that M1 macrophages are strongly activated in obesity. Metabolically, M2 macrophages are more dependent on oxidative phosphorylation, the dramatic switch to which results in phenotypic and functional changes in macrophages [31]. Moreover, upregulated genes in HFD mice indicated activation of microphages as well as dysregulation of macrophage functions. Similar to this, chronic expression of IFNγ in ARE-Del^-/-^ mice induced abnormal macrophage function, such as defective autophagy, which has been linked to dysregulated lipid metabolism through altered NR signaling [19]. The macrophage is a primary cell type in the formation of most granulomas, where immune cell aggregates form in response to chronic inflammatory stimuli such as *Mycobacterium tuberculosis* infection [32]. Considering that granuloma formation is a pathophysiological phenotype in both PBC and ARE-Del^-/-^ mice, these data suggest that dysregulated lipid metabolism in macrophages via altered NR signaling pathways may play a critical role in granuloma formation as well as obesity-associated pathology.

One of the most recognized adipokines, leptin, is a key molecule in the interplay between energy metabolism homeostasis and metabolism-linked inflammatory responses. Leptin is involved in the JAK/STAT pathway along with IFNγ, and acts synergistically with IFNγ-mediated inflammatory responses [33]. In contrast, alternative p38, ERK, JNK, PKC, and PI3K/AKT pathways overlap in signaling via both leptin and IFNγ receptors [34,35,36,37]. IFNγ binding to its specific receptor activates JAK1/JAK2, resulting in dimerization of STAT1, which acts as a transcription factor to induce IFNγ response genes. In contrast, leptin binding to its receptor induces phosphorylation of JAK2, which subsequently activates STAT3 via dimerization.

Given that JAK2 plays a critical role in brown adipose tissue function and diet-induced thermogenesis in HFD mice [38], JAK2 signaling between leptin and IFNγ may play a critical role in obesity-related inflammation and metabolic disorders. Dimerized STAT3 acts as a transcription factor and upregulates *SOCS3* expression, which can induce hypothalamic leptin resistance linked to obesity [39,40]. As we demonstrated previously, female ARE-Del^-/-^ mice specifically induced *SOCS3* gene expression in the liver, inhibition of which correlates with the activation of alternative pathways, such as PI3K/AKT [41]. It has also been reported that IFNγ induces *SOCS3* expression, which further inhibits STAT3 phosphorylation in endothelial cells, implying that IFNγ can desensitize liver cells to leptin signaling via the SOCS3-mediated inhibition of STAT3 [42]. Taken together, the signaling interaction between leptin and IFNγ, particularly in adipose tissue of obese individuals, may play a key modulating role in obesity-associated metabolic disorders and development of pathology.

Here, we demonstrated a potential therapeutic effect of luteolin in the early pathogenic development of PBC, as well as in other Th1-mediated autoimmune diseases. Luteolin (3′,4′,5,7-tetrahydroxyflavone) has been thought to have beneficial anti-cancer, anti-oxidant, and anti-inflammatory effects [43,44,45]. However, the mechanisms by which these beneficial effects occur have not been elucidated. Luteolin inhibits antigen-specific IFNγ production in autoreactive T cells in vitro [46], indicating that luteolin has a suppressive effect on IFNγ production through the adaptive immune response. Our data support the suppressive role luteolin in IFNγ signaling, but also reveal that luteolin can suppress early IFNγ-induced innate immune responses, possibly via NR signaling, by particularly enhancing the PPAR signaling pathway. Luteolin not only suppressed MHC I and II class antigen presentation pathways, but also modulated FOA and Oxphos metabolic pathways, which are critical for functional and phenotypical changes in macrophages. This luteolin-mediated metabolic change may be a key regulatory mechanism to suppress early IFNγ-mediated inflammatory reaction in the livers of HFD mice.

Additionally, these data demonstrate that immune network-based analysis can efficiently integrate different platforms or technologies that measure gene expression analysis. Since RNA-sequencing emerged last decade as a more robust and reproducible analysis tool than previous commonly used gene expression arrays [47], it has often been required to compare gene expression profiles generated by these two different technologies. However, comparison of differentially expressed genes (DEGs) identified in these different platforms is not possible, as the datasets are incompatible with each other, even when generated from the same biological condition. Given that immune responses are prompt, and gene sets are simultaneously activated, immune network-based analysis can be consistent and independent of different gene expression analysis platforms. Therefore, this methodological approach can be further used for the analysis of immune responses to compare mechanisms as well as the drug repurposing. Immune network-based analysis, as shown here, was used to predict the therapeutic effect of luteolin in the early stages of PBC pathogenesis.

## 4. Materials and Methods 

### 4.1. Data Source

GSE76309 and GSE54189 datasets were obtained from the GEO database (http://www.ncbi.nlm.nih.gov/geo/). In the GSE76309 dataset [6], mRNA was isolated from the liver of ARE-Del^-/-^ mice and analyzed by Illumina HiSeq2500 sequencing with TruSeq v4 chemistry. BAM files were generated by Tophat (v2.0.8), aligned to the mouse reference genome mm9 and ensemble v70 transcripts. Each BAM file was uploaded into Partek Genome Studio 6.6 to identify DEGs. The GSE54189 dataset [15] includes gene expression profiles of adipose tissue and liver from C57BL/6 mice, the same genetic background as ARE-Del^-/-^ mice [24]. This dataset contains array-based gene expression analyses using Illumina Mouse WG-6 v2 Expression BeadChips (Illumina, San Diego, CA, USA). Further data analysis was performed with ArrayAssist software (Stratagene, La Jolla, CA, USA) using Bioconductor.

### 4.2. Gene Set Enrichment and Pathway Analysis

Although GSEA and IPA are commonly used to characterize biological functions of gene expression profiles, the difficulty lies in selecting the appropriate comparison dataset and pathways from among an ever-increasing collection of datasets. As immune responses are often dynamically regulated by posttranslational modification [48,49], knowledge-based analysis is essential in order to characterize the immune network in human diseases as performed in this research. For GSEA, we downloaded target gene sets from the Molecular Signatures Database (MSigDB). The ranked experimental gene was listed along with the enriched gene sets. For IPA, statistically significant DEGs were tested using knowledge-based datasets. Z-score, ratio, and *p*-value were calculated to define the significance of a gene functioning in a network, as well as intergenic relationships. *p*-value was calculated using the right-tailed Fisher’s exact test, and a *p*-value less than 0.05 signified statistical significance and nonrandom association.

## 5. Conclusions

These data provide new insights into the mechanistic understanding of obesity-associated inflammatory responses in terms of the IFNγ signaling pathway and the etiological link to pathological development of PBC. Furthermore, we present a potential therapeutic application of luteolin in the treatment of PBC, as well as other Th1-mediated autoimmune diseases, based on comparison of similar immune responses.

## Figures and Tables

**Table 1:** The top upstream transcriptional regulators in high-fat-diet mice, determined by the Ingenuity^®^ Knowledge Base.

	Top Regulators	*p*-Value
1	IFNγ	9.19 × 10^−24^
2	TLR4	5.43 × 10^−23^
3	TNF	1.04 × 10^−21^

Fisher’s exact test *p*-values are calculated based on the overlap between the dataset genes and genes regulated by the appropriate transcriptional regulator.

**Table 2:** Suppressive effects of luteolin treatment on the top 20 altered pathways in high-fat-diet mice.

	Top 20 Canonical Pathways in HFD mice	HFD	ARE	Lut
1	Fcγ Receptor-Mediated Phagocytosis in Macrophages and Monocytes	up	up	down
2	Leukocyte Extravasation Signaling	up	up	down
3	Phagosome Formation	up	up	down
4	Granulocyte Adhesion and Diapedesis	up	up	down
5	Natural Killer Cell Signaling	up	up	down
6	IL-10 Signaling	up	up	-
7	Dendritic Cell Maturation	up	up	down
8	Atherosclerosis Signaling	up	up	down
9	Production of Nitric Oxide and Reactive Oxygen Species in Macrophages	up	up	down
10	PI3K Signaling in B Lymphocytes	up	up	-
11	TREM1 Signaling	up	up	down
12	Hepatic Fibrosis/Hepatic Stellate Cell Activation	up	up	down
13	Altered T cell and B cell Signaling in Rheumatoid Arthritis	up	up	down
14	Agranulocyte Adhesion and Diapedesis	up	up	down
15	Communication between Innate and Adaptive Immune Cells	up	up	down
16	CCR5 Signaling in Macrophages	up	up	down
17	Role of Pattern Recognition Receptors in Recognition of Bacteria and Viruses	up	up	down
18	Toll-like Receptor Signaling	up	up	down
19	Role of NFAT in Regulation of the Immune Response	up	up	down
20	B Cell Receptor Signaling	up	up	down

HFD, high-fat-diet mice; ARE, ARE-Del^-/-^ mice; Lut, HFD with luteolin treatment.

## Abbreviations

ARE	AU-rich element
DEG	Differentially expressed genes
FAO	Fatty acid oxidation
HFD	High-fat diet
IPA	Ingenuity pathway analysis
GSEA	Gene set enrichment analysis
IFNγ	Interferon gamma
Oxphos	Oxidative phosphorylation
PBC	Primary biliary cholangitis
RA	Rheumatoid arthritis
MGI	Mouse genome informatics
NK	Natural killer
NR	Nuclear receptor
SLE	Systemic lupus erythematosus
Ub	Ubiquitin
UBC	Ubiquitin C
